# Vasodilator-stimulated phosphoprotein (VASP) is not a major mediator of platelet aggregation, thrombogenesis, haemostasis, and antiplatelet effect of prasugrel in rats

**DOI:** 10.1038/s41598-018-28181-8

**Published:** 2018-07-02

**Authors:** Yusuke Ito, Kousaku Ohno, Yuka Morikawa, Atsuyuki Tomizawa, Makoto Mizuno, Atsuhiro Sugidachi

**Affiliations:** Rare Disease & LCM Laboratories, Daiichi Sankyo Co., Ltd, 1-2-58, Hiromachi, Shinagawa-ku, Tokyo 140-8710 Japan

## Abstract

Vasodilator-stimulated phosphoprotein (VASP) is a member of actin regulatory proteins implicated in platelet adhesion. In addition, phosphorylation of VASP is utilised for the assessment of platelet reactivity in patients treated with P2Y_12_ receptor antagonists, a class of antiplatelet agents. However, the role of VASP in platelet aggregation, thrombogenesis, haemostasis, and the antiplatelet effect of P2Y_12_ receptor antagonists remains unclear. We investigated these effects using heterozygous and homozygous VASP knockout rats generated with a CRISPR/Cas9 system. Baseline characteristics, such as haematology and other biochemical parameters, were comparable among the genotypes. *In vitro* platelet aggregation stimulated by adenosine diphosphate (ADP) or collagen, P-selectin expression of rat platelets treated with ADP, and *in vivo* thrombocytopenia induced by collagen were also comparable among the genotypes. In addition, *in vivo* thrombogenesis in a ferric chloride-induced arterial thrombosis model and bleeding time were also comparable among the genotypes. Furthermore, the *in vitro* antiplatelet effect of prasugrel, a third-generation P2Y_12_ receptor antagonist, was unaffected by VASP knockout. Although phosphorylated VASP is still an important surrogate marker specific for P2Y_12_ antagonists, our findings demonstrate that VASP is not a major mediator of platelet aggregation, thrombogenesis, haemostasis, and the antiplatelet effect of prasugrel in rats.

## Introduction

Coronary artery disease (CAD) is the leading cause of death and disability in developed countries^1^. Acute coronary syndrome (ACS) is a frequent life-threatening manifestation of CAD due to decreased blood flow caused by thrombi in the coronary arteries^2^. Dual-antiplatelet therapy combined with aspirin and an antagonist of P2Y_12_ receptor, an adenosine diphosphate (ADP) receptor expressed on platelets, is the standard treatment for patients with ACS or who are undergoing scheduled percutaneous coronary intervention to prevent platelet aggregation and reduce cardiovascular events^3^.

Monitoring of antiplatelet effects in patients treated with such antiplatelet agents is important for appropriate management of the disease and understanding of the drug’s efficacy and safety. Light transmission aggregometry (LTA) has been widely used to assess platelet function^4^, but the data obtained by LTA include the contribution of P2Y_1_, another ADP receptor on platelets^5^, as well as P2Y_12_. Because thienopyridines (clopidogrel and prasugrel) and ticagrelor are known to antagonise P2Y_12_ receptor specifically^6–9^, the platelet reactivity index (PRI) calculated from phosphorylated vasodilator-stimulated phosphoprotein (VASP) is used for assessment of P2Y_12_-selective antagonism^10,11^.

VASP is a member of a conserved family of actin regulatory proteins that is implicated in actin-based processes, such as filopodia formation and adhesion of platelets^12,13^. Upon stimulation with ADP, cyclic AMP (cAMP) in platelets decreased via P2Y_12_ receptor-Gi proteins function and consistently VASP Ser157 phosphorylation also decreased^14,15^. Platelets derived from VASP knockout mice showed exaggerated fibrinogen binding when stimulated with collagen and thrombin, and this exaggerated binding was partially mediated by cyclic guanosine monophosphate (cGMP)/cAMP which activate cyclic nucleotide-dependent kinases^16,17^, suggesting its involvement in physiological platelet functions. In addition, previous *ex vivo* studies revealed that platelets from VASP knockout mice significantly adhered and/or tethered to injured and/or denuded endothelia in NO-dependent manner^17^, suggesting its significance on pathophysiological platelet functions. Interestingly, tail bleeding time was comparable between wild-type mice and VASP knockout mice^16^. However, direct involvement of VASP in ADP-induced platelet activation and aggregation has not yet been documented. In addition, the role of VASP in *in vivo* platelet aggregation and thrombogenesis awaits further clarification. It is also unknown whether VASP is essentially involved in the antiplatelet effect of P2Y_12_ receptor antagonists.

We created VASP knockout rats and investigated whether VASP was directly involved in platelet activation, aggregation, thrombogenesis, and haemostasis. In addition, the role of VASP in the antiplatelet effect of prasugrel, a third-generation thienopyridine prodrug, was also explored.

## Results

### Generation of VASP knockout rats

**Figure 1:**
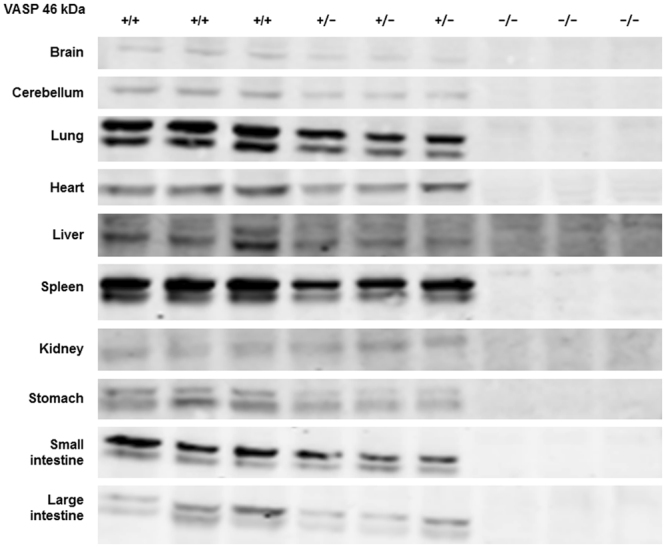
Vasodilator-stimulated phosphoprotein (VASP) expression in VASP^+/+^, VASP^+/−^, and VASP^−/−^ rats. Sodium dodecyl sulfate polyacrylamide gel electrophoresis (SDS-PAGE) and Western blot analysis were performed with 10 μg of protein extracted from each organ. The primary antibody used for VASP was VASP antibody (3112 S, Cell Signaling Technology, Inc.) with 1,000-fold dilution. *n* = 3 per genotype. +/+, wild type; +/−, heterozygous VASP knockout; −/−, homozygous VASP knockout.

Founder rats (41 of 91 neonates) were confirmed by polymerase chain reaction (PCR) screening (Supplementary Fig. S1). Ten of the 41 founders were subjected to direct sequencing, and 9 of the 10 subjects confirmed successful nucleotide deletion encoding the VASP gene. Four lines of the deletion mutant were systematised, and F1 generations were confirmed without any off-target deletions predicted from the guide RNA sequences (5ʹ-side: Rasal1, Msto1, Eml1, Fbxl1, and Tp53inp2; 3ʹ-side: Gimap8, Tmco4, and RGD1564380) in the F1 generation. Body weight at 7 to 8 weeks of age was 220.3 ± 4.2 g in wild-type (VASP^+/+^) rats, 214.4 ± 8.0 g in heterozygous VASP knockout (VASP^+/−^) rats, and 226.4 ± 8.2 g in homozygous VASP knockout (VASP^−/−^) rats. Western blot analysis of various organs revealed global VASP deletion in VASP^−/−^ rats (Fig. 1), whereas β-actin expression was confirmed in VASP^+/+^, VASP^+/−^, and VASP^−/−^ rats (Supplementary Fig. S2).

### Effects of VASP deletion on haematology and blood biochemistry

The effects of VASP deletion on haematological and blood biochemical parameters are summarised in Tables 1 and 2. Most of the parameters tested were comparable among genotypes (Table 1). Platelet counts (×10^3^/μL) in VASP^+/+^, VASP^+/−^, and VASP^−/−^ rats were 1,149 ± 46, 1,176 ± 17, and 1,078 ± 39 (*P* < 0.05, VASP^−/−^ vs. VASP^+/+^), respectively. Eosinophil counts (×10^3^/μL) in VASP^+/+^, VASP^+/−^, and VASP^−/−^ rats were 0.7 ± 0.0, 1.0 ± 0.1, and 0.7 ± 0.1 (*P* < 0.05, VASP^+/−^ vs. VASP^−/−^), respectively.

**Table 1:** Haematological analysis of vasodilator-stimulated phosphoprotein (VASP) knockout rats.

		Genotype		
		VASP^+/+^	VASP^+/−^	VASP^−/−^
RBC (×10^6^/μL)		7.51 ± 0.12	7.56 ± 0.16	7.64 ± 0.15
Hb (g/dL)		13.9 ± 0.2	14.0 ± 0.2	14.1 ± 0.3
Ht (%)		44.3 ± 0.6	44.6 ± 0.6	45.0 ± 0.7
MCV (fL)		59.0 ± 0.4	59.1 ± 0.6	58.9 ± 0.4
MCH (pg)		18.6 ± 0.1	18.5 ± 0.2	18.5 ± 0.1
MCHC (g/dL)		31.4 ± 0.2	31.3 ± 0.1	31.4 ± 0.3
WBC (×10^3^/μL)		5.50 ± 0.32	5.06 ± 0.28	4.68 ± 0.34
	Bas (%)	0.1 ± 0.0	0.1 ± 0.0	0.1 ± 0.0
	Eos (%)	0.7 ± 0.0	1.0 ± 0.1^†^	0.7 ± 0.1
	Neut (%)	13.7 ± 0.6	13.9 ± 0.8	14.6 ± 0.9
	Lym (%)	83.6 ± 0.6	83.2 ± 0.7	82.7 ± 1.0
	Mon (%)	1.4 ± 0.2	1.2 ± 0.1	1.4 ± 0.1
	Others (%)	0.6 ± 0.1	0.5 ± 0.0	0.5 ± 0.1
Platelets (×10^3^/μL)		1149 ± 46	1176 ± 17	1078 ± 39*
Reticulocyte	%	5.54 ± 0.18	5.40 ± 0.17	5.05 ± 0.29
	×10^3^/L	415.3 ± 11.0	407.9 ± 15.1	383.2 ± 15.0
PT (s)		10.0 ± 0.1	10.2 ± 0.1	10.3 ± 0.1
aPTT (s)		14.0 ± 0.3	13.8 ± 0.3	14.0 ± 0.6

Data represent means ± SEM (*n* = 6). Statistical analyses were performed with Student’s *t*-test (VASP^+/+^ vs. VASP^−/−^ as a primary analysis and VASP^+/−^ vs. VASP^−/−^ as a subanalysis). ^*^P < 0.05 vs. VASP^+/+^. ^†^*P* < 0.05 vs. VASP^−/−^. RBC, red blood cells; Hb, haemoglobin; Ht, haematocrit; MCV, mean corpuscular volume; MCH, mean corpuscular haemoglobin; MCHC, mean corpuscular haemoglobin concentration; WBC, white blood cells; Bas, basophils; Eos, eosinophils; Neut, neutrophils; Lym, lymphocytes; Mon, monocytes; PT, prothrombin time; aPTT, activated partial thromboplastin time.

**Table 2:** Blood biochemistry in vasodilator-stimulated phosphoprotein (VASP) knockout rats.

	Genotype		
	VASP^+/+^	VASP^+/−^	VASP^−/−^
GOT (IU/L)	72 ± 2	74 ± 2	72 ± 3
GPT (IU/L)	45 ± 3	53 ± 4	49 ± 2
ALP (IU/L)	1171 ± 42	1132 ± 82	1023 ± 53
TG (mg/dL)	53 ± 6	47 ± 3	47 ± 5
TC (mg/dL)	79 ± 4	75 ± 2	74 ± 3
BUN (mg/dL)	15.4 ± 0.9	15.3 ± 0.8	18.9 ± 3.2
CRE (mg/dL)	0.24 ± 0.01	0.25 ± 0.01	0.25 ± 0.01
TB (mg/dL)	0.00 ± 0.00	0.00 ± 0.00	0.00 ± 0.00
TP (g/dL)	5.5 ± 0.0	5.6 ± 0.1	5.5 ± 0.1
Alb (g/dL)	3.2 ± 0.0	3.2 ± 0.0	3.3 ± 0.0
Glu (mg/dL)	123 ± 6	117 ± 5^†^	136 ± 4
IP (mg/dL)	8.1 ± 0.2	7.8 ± 0.1	8.0 ± 0.2
Ca (mg/dL)	10.2 ± 0.1	10.2 ± 0.1	10.3 ± 0.1
Na (mEq/L)	139.9 ± 0.7	139.8 ± 0.5	140.3 ± 0.4
K (mEq/L)	4.98 ± 0.31	5.09 ± 0.23	4.99 ± 0.22
Cl (mEq/L)	103.3 ± 0.3	103.8 ± 0.3	103.5 ± 0.6

Data represent means ± SEM (*n* = 6). Statistical analyses were performed with Student’s *t*-test (VASP^+/+^ vs. VASP^−/−^ as a primary analysis and VASP^+/−^ vs. VASP^−/−^ as a subanalysis). ^†^*P* < 0.05 vs. VASP^−/−^. GOT, glutamate oxaloacetate transaminase; GPT, glutamate pyruvate transaminase; ALP, alkaline phosphatase; TG, triacylglycerol; TC, total cholesterol; BUN, blood urea nitrogen; CRE, creatinine; TB, total bilirubin; TP, total protein; Alb, albumin; Glu, glucose; IP, inorganic phosphate; Ca, calcium; Na, sodium; K, potassium; Cl, chloride.

### Effects of VASP deletion on platelet aggregation and P-selectin expression

The effects of VASP deletion on platelet aggregation and activation induced by ADP and collagen are summarised in Table 3. Statistically significant differences in ADP- or collagen-induced platelet aggregation were not observed. P-selectin expression was significantly increased by ADP treatment compared with phosphate-buffered saline (PBS) treatment in each genotype. In the PBS-treated group, the number of P-selectin-positive cells was lower in VASP^−/−^ rats than in VASP^+/+^ rats (*P* < 0.05). Among rats treated with 1.25 or 5 μmol/L of ADP, the number of P-selectin-positive cells was lower in VASP^−/−^ rats than in VASP^+/−^ rats (*P* < 0.05).

**Table 3:** Platelet aggregation and platelet P-selectin expression in vasodilator-stimulated phosphoprotein (VASP) knockout rats.

		Genotype		
		VASP^+/+^	VASP^+/−^	VASP^−/−^
Platelet aggregation (%)				
ADP (μmol/L)	1.25	3 ± 1	3 ± 0	3 ± 1
	5	30 ± 2	29 ± 1	28 ± 2
	20	46 ± 2	48 ± 2	48 ± 1
Collagen (μg/mL)	2	4 ± 0	4 ± 1	4 ± 0
	10	65 ± 3	67 ± 0	66 ± 3
P-selectin expression (arbitrary units)				
ADP (μmol/L)	PBS	181 ± 1	180 ± 3	170 ± 3^†^
	1.25	247 ± 5**	277 ± 10**^‡^	234 ± 7**
	5	376 ± 7**	425 ± 19**^‡^	371 ± 14**
	20	496 ± 11**	556 ± 26**	490 ± 17**

Adenosine diphosphate (ADP)- and collagen-induced platelet aggregation of platelet-rich plasma and ADP-induced P-selectin expression in CD61-positive cells were evaluated. Data represent means ± SEM (*n* = 6). Statistical analyses were performed with Dunnett’s multiple comparison test (phosphate-buffered saline [PBS] vs. ADP-treated groups) and Student’s *t*-test (VASP^+/+^ vs. VASP^−/−^ as a primary analysis and VASP^+/−^ vs. VASP^−/−^ as a subanalysis). ^**^P < 0.01 vs. vehicle (Dunnett’s multiple comparison test). ^†^*P* < 0.05 vs. VASP^+/+^ (Student’s *t*-test). ^‡^*P* < 0.05 vs. VASP^−/−^ (Student’s *t*-test).

### Effects of VASP deletion on collagen-induced thrombocytopenia *in vivo*

**Figure 2:**
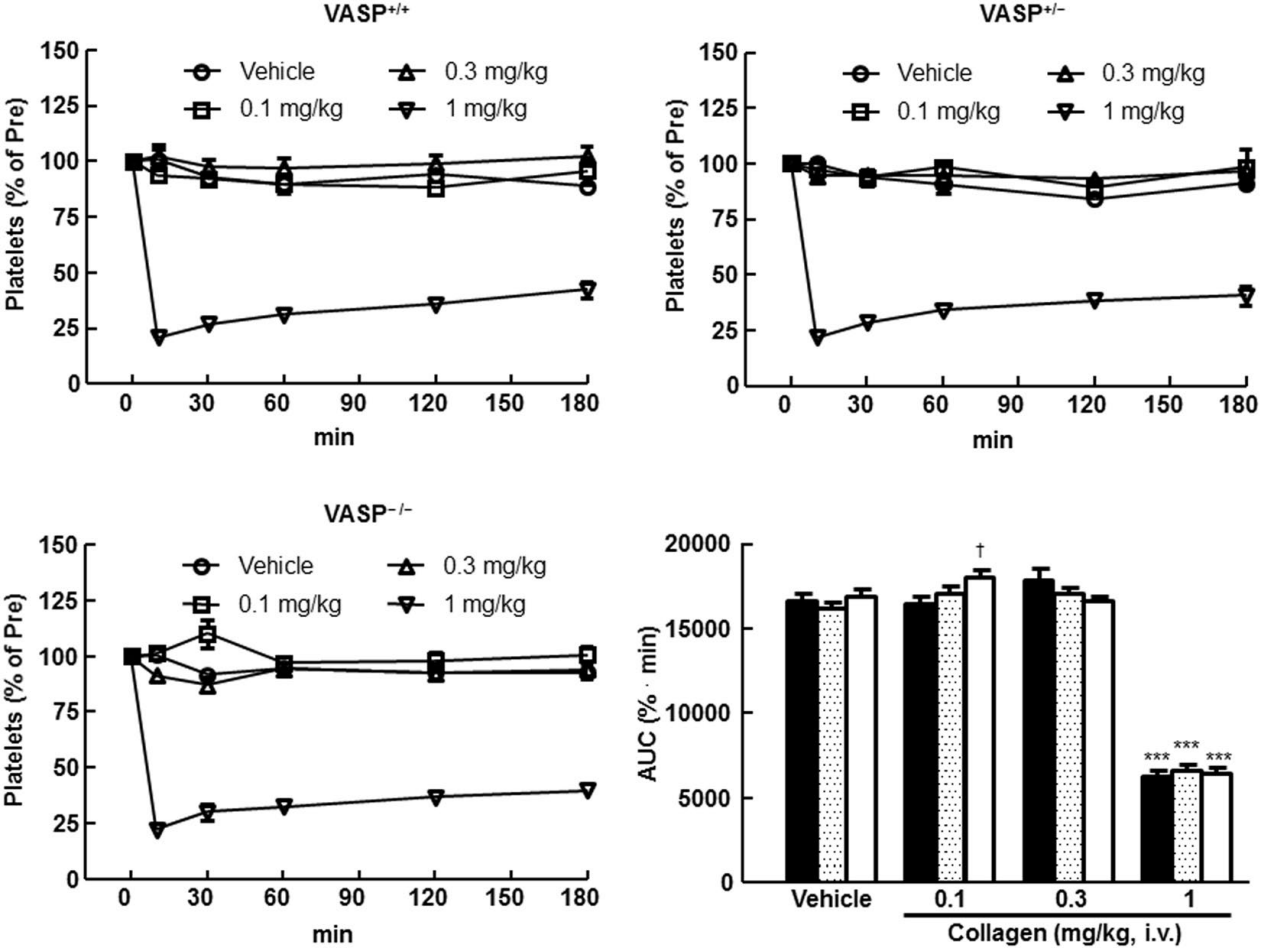
Impact of vasodilator-stimulated phosphoprotein (VASP) deficiency on collagen-induced thrombocytopenia in rats. Closed bars: VASP^+/+^; dotted bars: VASP^+/−^; open bars: VASP^−/−^. Data are means ± SEM (*n* = 4 or 5). ***P < 0.0001 vs. vehicle (Dunnett’s multiple comparison test). †*P* < 0.05 vs. VASP^+/+^ (Student’s *t*-test). AUC, area under the curve.

Changes in platelet count and their area under the curve (AUC) are presented in Fig. 2. Time-dependent changes in platelet count were similar regardless of genotype. The AUC (% · min) in rats treated with 0.1 mg/kg of collagen was 16,434.0 ± 465.4 (VASP^+/+^), 17,036.1 ± 478.5 (VASP^+/−^), and 18,046.4 ± 425.3 (VASP^−/−^). A statistically significant increase in AUC was observed in VASP^−/−^ rats compared with VASP^+/+^ rats (*P* < 0.05). There were no statistically significant differences in AUC among rats of different genotypes treated with other collagen doses.

### Effects of VASP deletion on thrombogenesis

**Figure 3:**
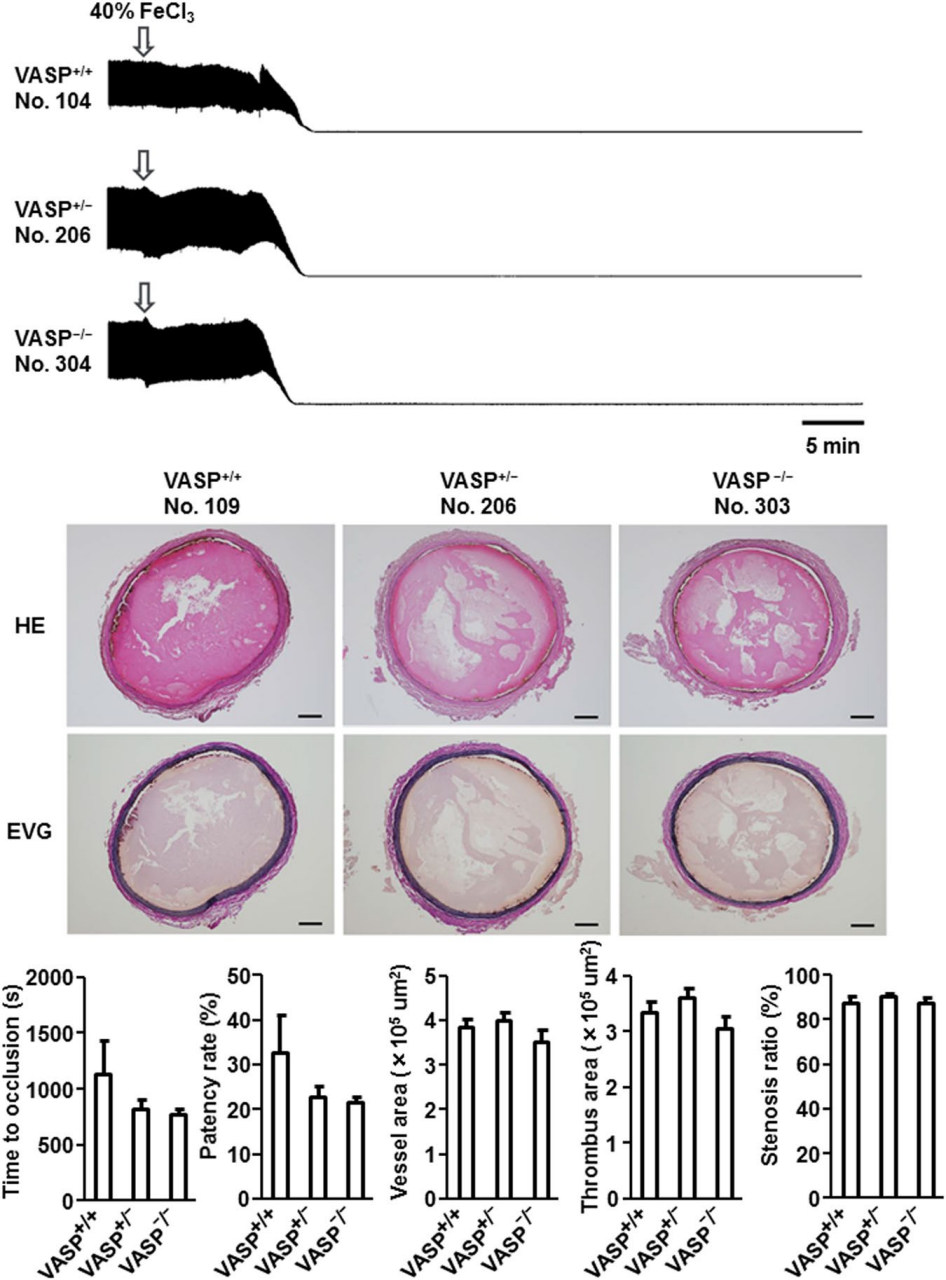
Effect of vasodilator-stimulated phosphoprotein (VASP) knockout on thrombus formation in FeCl_3_-induced arterial thrombosis in rats. Typical tracings of blood flow, haematoxylin and eosin (HE), and Elastica van Gieson (EVG) are presented for each genotype. Summary statistics of time in seconds to first occlusion (TTO), patency rate, vessel area, thrombus area, and stenosis ratio are also presented. Data are means ± SEM (*n* = 10).

The effects of VASP deletion on thrombus formation in the ferric chloride (FeCl_3_)-induced carotid artery thrombosis model are summarised in Fig. 3. Blood flow was stopped immediately after FeCl_3_ application. Cross sections of the injured carotid artery stained with haematoxylin and eosin (HE) and Elastica van Gieson (EVG) revealed the presence of large thrombi regardless of VASP genotype. There were no statistically significant differences among rats of different genotypes in the time in seconds to first occlusion (TTO), patency rate, or morphometric parameters, including vessel area, thrombus area, and stenosis ratio.

### Effects of VASP deletion on haemostasis

The effect of VASP gene deletion on haemostasis was evaluated in a tail bleeding model. There were no statistically significant differences among rats of different genotypes in bleeding time: 269 ± 17 s (VASP^+/+^), 231 ± 12 s (VASP^+/−^), and 272 ± 16 s (VASP^−/−^).

### Effects of VASP deletion on PRI

**Figure 4:**
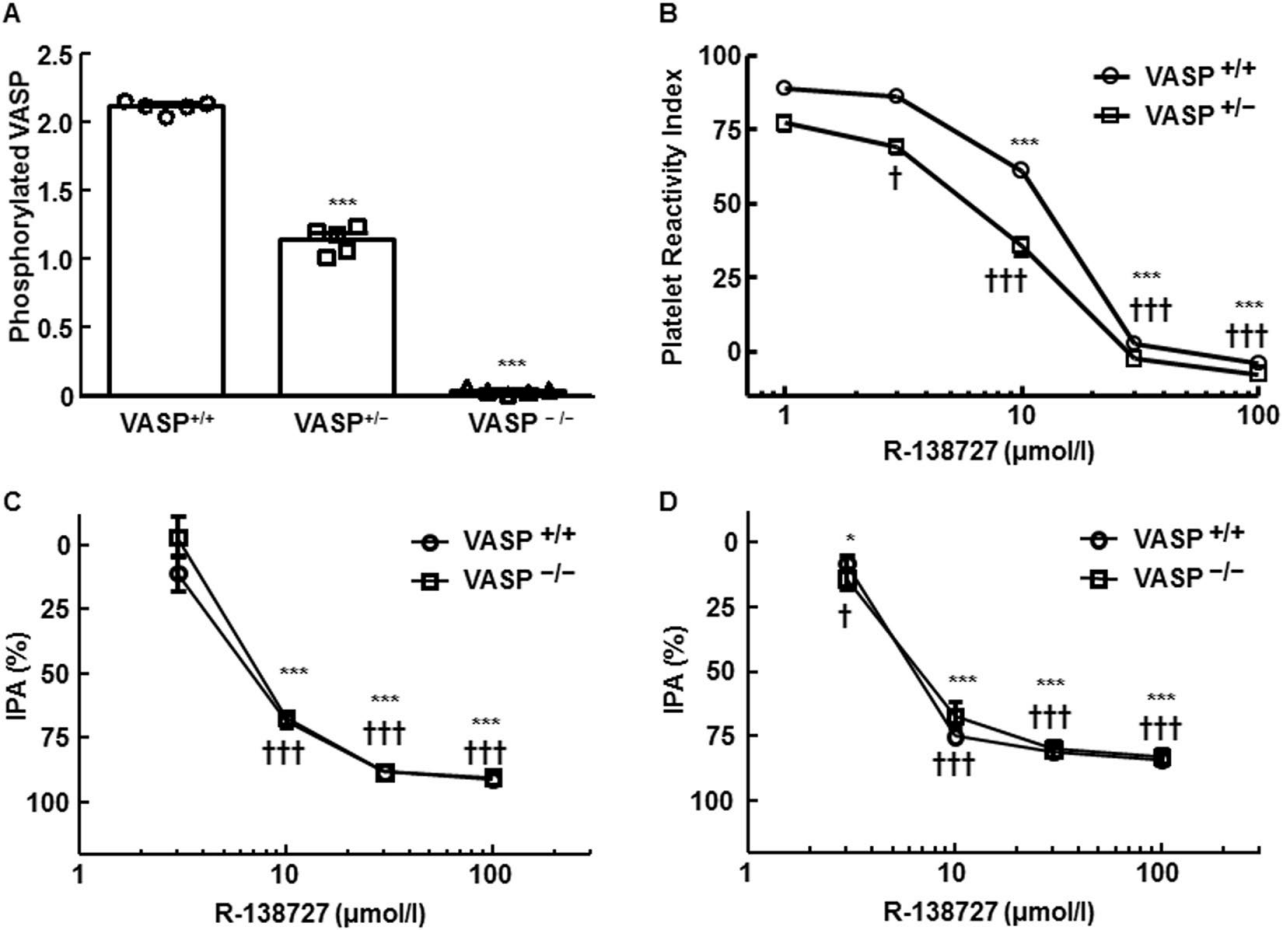
Influence of vasodilator-stimulated phosphoprotein (VASP) knockout on phosphorylated VASP antigen, platelet reactivity index (PRI), and inhibition of platelet aggregation (IPA) in rats. (**A**) Optical density at 450 nm reflecting quantity of phosphorylated VASP antigen in VASP^+/+^, VASP^+/−^, and VASP^−/−^ rats. Data are means ± SEM (*n* = 5). ***P < 0.0001 vs. VASP^+/+^. (**B**) Effect of R-138727 on platelet reactivity index in VASP^+/+^ and VASP^+/−^ rats. Data are means ± SEM (*n* = 5). ***P < 0.0001 vs. vehicle in VASP^+/+^ rats. ^†^*P* < 0.05, ^†††^*P* < 0.0001 vs. vehicle in VASP^+/−^ rats. (**C,D**) Effect of R-138727 on ADP- or collagen-induced platelet aggregation in VASP^+/+^ and VASP^−/−^ rats. Data are means ± SEM (*n* = 5). *P < 0.05, ***P < 0.0001 vs. vehicle in VASP^+/+^ rats. ^†^*P* < 0.05, ^†††^*P* < 0.0001 vs. vehicle in VASP^−/−^ rats.

The effects of VASP deletion on PRI were investigated. The quantity of phosphorylated VASP in blood (optical density at 450 nm) was 2.126 ± 0.020 (VASP^+/+^), 1.148 ± 0.043 (VASP^+/−^), and 0.041 ± 0.009 (VASP^−/−^) (Fig. 4A). Statistically significant decreases were observed in VASP^+/−^ and VASP^−/−^ rats compared with VASP^+/+^ rats (*P* < 0.0001). Figure 4B shows the PRI of R-138727-treated whole blood from VASP^+/+^ and VASP^+/−^ rats. PRI calculation was not performed on VASP^−/−^ rats because their optical density values were almost negligible. Dose-dependent, statistically significant (*P* < 0.0001) decreases in PRI were observed in rats of both genotypes. The 50% inhibitory concentration (IC_50_) values and their 95% confidence intervals (95% CIs) of R-138727 on PRI in VASP^+/+^ and VASP^+/−^ rats were 10.81 (8.51 to 13.79) μmol/L and 7.75 (6.41 to 9.32) μmol/L, respectively.

### Effects of VASP deletion on antiplatelet effect of R-138727

To assess whether VASP is involved in the antiplatelet effect of prasugrel, inhibition of platelet aggregation (IPA) of R-138727 was compared between VASP^+/+^ and VASP^−/−^ rats (Fig. 4C,D). R-138727 inhibited platelet aggregation in platelet-rich plasma induced by 20 μmol/L of ADP or 10 μg/mL of collagen. The IC_50_ values against each agonist were equivalent in rats of the two genotypes: 6.8 μmol/L (95% CI, 5.7–8.6) in VASP^+/+^ rats and 7.5 μmol/L (95% CI, 6.4–9.2) in VASP^−/−^ rats for ADP-induced aggregation; 6.4 μmol/L (95% CI, 5.9–6.9) in VASP^+/+^ rats and 7.5 μmol/L (95% CI, 5.7–8.3) in VASP^−/−^ rats for collagen-induced aggregation.

## Discussion

VASP knockout mice previously generated by target disruption of exons 2 to 13 had normal life spans, no obvious tissue abnormalities, and platelet numbers comparable with those of wild-type mice^16^. There are no previous reports on VASP knockout rats. Because rats are larger than mice, they have several experimental advantages over mice for investigation of more elaborate thrombotic disease models. In addition, CRISPR/Cas9-mediated genome editing produces specific deletion of whole coding regions of genes of interest in any animal species^18^. Therefore, we thought that VASP knockout rats would offer further insight into VASP function in research on thrombosis and haemostasis.

The present study investigated the effects of VASP on platelet aggregation, thrombogenesis, haemostasis, and the antiplatelet effect of prasugrel in rats. CRISPR/Cas9-mediated VASP knockout rats were successfully generated without off-target gene deletion or critical changes in blood coagulation parameters, including prothrombin time and activated partial thromboplastin time, which are important for the evaluation of thrombus formation and haemostasis. In addition, other biochemical parameters, such as triacylglycerol and total cholesterol, which may affect platelet aggregation and thrombogenesis^19–21^, were not changed, in spite of VASP deficiency. The reasons for statistically significant differences between rats of different genotypes in platelet count (VASP^−/−^ < VASP^+/+^), eosinophil count (VASP^−/−^ < VASP^+/−^), and plasma glucose (VASP^+/−^ < VASP^−/−^) are unclear, but we regarded the differences as negligible. These results suggest that VASP knockout rats suitable for our research objective were successfully generated.

Rats homozygous for VASP deficiency did not differ from rats with wild-type VASP in ADP- or collagen-induced platelet aggregation and ADP-induced expression of platelet P-selectin. These results suggest that VASP has no major effects on ADP- or collagen-induced platelet aggregation and activation *in vitro*. The finding of no impact of VASP on collagen-induced platelet aggregation is in line with a previous observation in VASP knockout mice^16^, suggesting a dispensable or minor effect of VASP on ADP- and collagen-induced platelet aggregation. To the best of our knowledge, this is the first report investigating the involvement of VASP in ADP- and collagen-mediated platelet functions in rats.

VASP is expressed not only on platelets but also on other cells, such as endothelial cells and vascular smooth muscle cells^16,22^, that play crucial roles in thrombosis and haemostasis. To investigate whether VASP deletion affects platelet aggregation, thrombogenesis, and haemostasis *in vivo*, rat models of collagen-induced thrombocytopenia, FeCl_3_-induced arterial thrombosis, and tail bleeding were employed. In line with *in vitro* platelet aggregation, there were no essential changes in platelet consumption, thrombus formation, and bleeding time *in vivo*. These results clearly demonstrate that VASP does not have vital roles in thrombogenesis or haemostasis in rats.

It was reported that platelets derived from VASP-deficient mice showed enhanced platelet tethering and firm platelet adhesion to endothelium in a P-selectin- and/or glycoprotein IIbIIIa (GPIIbIIIa)-dependent manner compared with wild-type platelets^17^. This enhanced platelet adhesion is further exaggerated in the presence of thrombotic insults, such as ischaemia–reperfusion (I/R) injury and atherosclerosis^17^. These discrepancies in VASP-mediated platelet phenotypes between previous findings and our findings might be due to species differences (mice vs. rats) and/or subjected thrombotic stimuli (I/R or atherosclerosis vs. FeCl_3_). Further analysis is required for better understanding of the role of VASP in platelet function and thrombosis in other contexts, such as stroke and balloon injury, in combination with other experimental approaches, including morphological and histological analysis of platelets and thrombi.

PRI is a biomarker for P2Y_12_-specific antagonism and is reported to be a good predictive marker for clinical outcomes in non-ST-elevation ACS^10,11,23^. In the present study, R-138727, an active metabolite of prasugrel, produced a concentration-dependent reduction in PRI in VASP^+/+^ rats, but no reduction in PRI was detected in VASP^−/−^ rats because of the absence of phosphorylated VASP antigen. On the other hand, the *in vitro* IC_50_ values of R-138727 on ADP- and collagen-induced platelet aggregation were comparable between VASP^+/+^ and VASP^−/−^ rats. These results demonstrated that VASP does not directly participate in the antiplatelet effect of prasugrel. At the same time, the IC_50_ values of PRI and IPA were comparable between VASP^+/+^ and VASP^−/−^ rats, indicating that PRI is a good biomarker reflecting P2Y_12_-mediated platelet inhibition in rats.

These data offer the novel finding that VASP does not play a causal role in platelet aggregation or the antiplatelet effect of prasugrel in rats. This finding also probably applies to other P2Y_12_ antagonists besides prasugrel, including clopidogrel and ticagrelor, although PRI is still an important surrogate marker specific for P2Y_12_ antagonists.

In conclusion, we show for the first time that VASP is not a major mediator of ADP- and collagen-induced platelet aggregation, thrombogenesis, haemostasis, and the antiplatelet effect of prasugrel, a third-generation thienopyridine P2Y_12_ antagonist, in rats. The results from VASP knockout rats may offer further insight into platelet and vascular biology.

## Methods

### Animal care

All experimental procedures were performed according to the in-house guidelines of the Institutional Animal Care and Use Committee of Daiichi Sankyo Co., Ltd. Animal health was monitored by animal care personnel twice a day on weekdays and once a day on weekends. Microbial monitoring was conducted with the use of sentinel animals once every 2 months. Proper care was taken or directed by attending veterinarians for animals showing abnormalities (distress in drinking water, feeding, breathing, or other abnormal behaviours such as self-injury or abnormal posture). All surgeries were performed with the animals under sodium thiopental anaesthesia (75 or 100 mg/kg, intraperitoneal [i.p.], Mitsubishi Tanabe Pharma Corporation) or isoflurane inhalation anaesthesia (3% to 5% for induction and 1% to 3% for maintenance, Pfizer, Inc.); all efforts were made to minimise suffering. The experimental protocols were approved by the Institutional Animal Care and Use Committee of Daiichi Sankyo (permit numbers: A1602079, A1700010, A1700011, A1700717, A1700718, and A1701247). Haematology, blood biochemistry, platelet aggregation, and thrombosis studies were conducted as approved by the Institutional Animal Experiment Committee of Nissei Bilis Co., Ltd. (permit numbers: 1701-02, 1705-01, and 1708-14). Flow cytometric analysis and bleeding assessment were conducted in compliance with the Guidance for Animal Care and Use of Ina Research, Inc. in accordance with the protocol (study numbers: IP16348 and IP17023) as approved by the Institutional Animal Care and Use Committee of Ina Research, Inc., which is fully accredited by AAALAC International (accredited unit number: 001107).

### Animals

VASP^+/−^ rats were generated and systematised using the CRISPR/Cas9 system^24^ at the Institute of Immunology Co., Ltd. In brief, guide RNAs were designed both upstream (5ʹ-GTGCAGCGTCCGAACCTCGC) and downstream (5ʹ-TAAATGCTATGCCCCATCGA) of the rat VASP gene. Fertilised eggs were collected from female Sprague–Dawley (SD) rats (Slc:SD, Japan SLC, Inc.) and were administered pregnant mare serum gonadotropin and human chorionic gonadotropin to induce superovulation for pronuclear injection. The guide RNAs and Cas9 mRNA were injected into the eggs, which were transplanted into pseudopregnant SD females to generate founder rats. The genotypes of the founder rats were analysed by PCR and direct sequencing of tail DNA samples to confirm gene deletion. The primer sequences were forward 5′-CCACAAGACTGAGAGGAAGGAGT and reverse 5ʹ-AGACACTGCAGGATGGAGGAAG. After germline transmission was confirmed, off-target gene deletion predicted from the guide RNA sequences (5ʹ-side: Rasal1, Msto1, Eml1, Fbxl1, and Tp53inp2; 3ʹ-side: Gimap8, Tmco4, and RGD1564380) (Supplementary Table S1) was analysed in the F1 generation. Primer sequences for the off-target analysis are listed in Supplementary Table S2. VASP^+/−^ parents were mated to generate VASP^+/+^, VASP^+/−^, and VASP^−/−^ rats. The rats were maintained in cages with three or fewer animals per cage with free access to chlorinated water and food (FR-2, Funabashi Farm Co., Ltd.). The conditions in the animal quarters were set at 23 °C ± 2 °C (allowable range: 18 °C to 28 °C), humidity 55% ± 10% (allowable range: 30% to 70%), and 12 h lighting cycle (lights on 7:00 to 19:00). The acclimation period was >3 days.

### Tested compounds

Prasugrel hydrochloride (prasugrel, CS-747S, molecular formula C_20_H_20_FNO_3_S·HCl) and its active metabolite R-138727 (molecular formula C_18_H_20_FNO_3_S) were supplied from Ube Industries, Ltd. Gum arabic (vehicle for prasugrel) was purchased from Wako Pure Chemical Industries, Ltd. Physiological saline (saline, vehicle for R-138727) was purchased from Otsuka Pharmaceutical Factory, Inc.

### Western blot analysis

The rats were anaesthetised with sodium thiopental (100 mg/kg, i.p.). Exsanguination was performed followed by perfusion of 150 mL of saline containing 2 U/mL of unfractionated heparin (Mochida Pharmaceutical Co., Ltd.) via the right ventricle with the use of a syringe pump (TE-351, Terumo Corporation) set at 1,200 mL/h to sacrifice the rats. The brain, cerebellum, lung, heart, liver, spleen, kidney, stomach, small intestine, and large intestine were immediately dissected and stored in RNAlater solution (Thermo Fisher Scientific, Inc.). Tissue homogenate was prepared with 20 mg of each organ in T-PER Tissue Protein Extraction Reagent (78510, Thermo Fisher Scientific, Inc.) in the presence of Halt Protease & Phosphatase Inhibitor Single-Use Cocktails (100×) (1861280, Thermo Fisher Scientific, Inc.). A handy homogenizer (Tissue Ruptor, Qiagen) was introduced to achieve rapid homogenisation. The supernatant was collected after centrifugation (20,400 × *g* for 10 min at 4 °C), and protein concentration was determined with a Pierce BCA Protein Assay Kit (23225, Thermo Fisher Scientific, Inc.). The tissue supernatant was added into the mixture of 4 × Laemli Sample Buffer (161-0747, Bio-Rad Laboratories, Inc.) and 2-mercaptoethanol (21418-42, Nacalai Tesque K.K.) in accordance with the manufacturer’s instructions. The samples were boiled for 5 min with a heat block set at 95 °C. The boiled samples (10 μg protein/lane for VASP and 1 μg protein/lane for β-actin) and molecular weight marker (Precision Plus Protein Dual Color Standards, 161-0374, Bio-Rad Laboratories, Inc.) were loaded onto 10% polyacrylamide gel (1706291, D.R.C. Co., Ltd.), and electrophoresis was performed with an electrophoresis tank connected to a power supply (KYOCA, D.R.C. Co., Ltd.). After electrophoresis, wet transfer from gel to polyvinylidene difluoride (PVDF) membranes was performed in NuPAGE transfer buffer (NP00061, Thermo Fisher Scientific, Inc.) for 120 min with an electrophoresis tank connected to a power supply (KYOCA-GP, D.R.C. Co., Ltd.) set at 250 mA. After transfer, the PVDF membranes were washed with TRIS-buffered saline + 0.1% Tween20 (TBS-T) and blocked with PVDF Blocking Reagent for Can Get Signal (Toyobo Co., Ltd.) for 1 h. Thereafter, the membranes were washed twice with TBS-T, primary antibodies (VASP antibody [3112 S, Cell Signaling Technology, Inc.] × 1/1,000 and monoclonal anti-β-actin antibody produced in mouse [A5441, Sigma-Aldrich Co. LLC] × 1/10,000) diluted with Can Get Signal Immunoreaction Enhancer Solution I (NKB-201, Toyobo Co., Ltd.) were added onto the membranes, and the membranes were incubated overnight at 4 °C. The membranes were washed three times with TBS-T, secondary antibodies (VASP: Alexa Fluor 680 anti-rabbit IgG or β-actin: Alexa Fluor 680 anti-mouse IgG [Thermo Fisher Scientific, Inc.] × 1/10,000) diluted with Can Get Signal Immunoreaction Enhancer Solution II (NKB-101, Toyobo Co., Ltd.) were added onto the membranes, and the membranes were incubated for another 1 h in the dark at room temperature. The membranes were washed three times with TBS-T, and protein was detected with an infrared imager (Odyssey, LI-COR, Inc.).

### Haematology and blood biochemistry

The rats were anaesthetised with isoflurane, and blood samples were withdrawn from the abdominal aorta for haematological and biochemical analyses. Haematological parameters were analysed with a haematology analyser (ADVIA2120, Siemens Healthcare K.K.). Blood clotting times were analysed with an automated coagulation analyser (CA-1500, Sysmex Corporation). Blood biochemistry was analysed with automated biochemical analysers (7180, Hitachi High-Technologies Corporation, and EA07, A&T Corporation).

### Platelet aggregation

Citrated blood was collected from the abdominal aorta and centrifuged to obtain platelet-rich plasma (PRP) (150 × *g*, 10 min at room temperature) and platelet-poor plasma (PPP) (2,000 × *g*, 10 min at room temperature). The platelet count in the PRP was determined, and the PRP was diluted with PPP to obtain a suspension of (500 ± 50) × 10^3^/μL platelets. The PRP (120 μL) was stirred for 1 min at 37 °C, and 5 μL of ADP (final concentration 1.25, 5, or 20 μmol/L) or collagen (final concentration 2 or 10 μg/mL) was added to induce platelet aggregation. Platelet aggregation was monitored for 15 min after addition of the agonist and recorded as maximum platelet aggregation with an automated platelet aggregometer (PRP31M3, IMI Corporation). In the evaluation of antiplatelet effect, R-138727 (1, 3, 10, 30, and 100 μmol/L) or vehicle (saline) was added to the PRP 30 min prior to addition of ADP (20 μmol/l) or collagen (10 μg/ml). Inhibition of antiplatelet effect (IPA) was calculated as

$${\rm{IPA}}\,( \% )=\frac{{\rm{A}}-{\rm{B}}}{{\rm{A}}}\times 100$$

where A = platelet aggregation (%) upon vehicle treatment and

B = platelet aggregation (%) upon R-138727 treatment.

### Flow cytometric analysis of P-selectin on platelets

P-selectin on platelets was measured as a platelet activation marker. Ten microliters of citrated whole blood was mixed with 3 μL of ADP (final concentration 1.25, 5, or 20 μmol/L) or PBS, 3 μL of fluorescein isothiocyanate (FITC)-labeled hamster anti-mouse CD61 antibody (Clone 2C9.G2, BD Biosciences), 3 μL of phycoerythrin (PE)-labeled mouse anti-human CD62P antibody (Clone Psel.KO.2.7, BD Biosciences), and 11 μL of PBS in a FACS tube. After 15 min at room temperature, 500 μL of FACS Lysing Solution (BD Biosciences) was added for 30 min to lyse and fix the blood cells. Analyses were performed with a FACS Canto II (BD Biosciences). FITC- and PE-positive cells were regarded as platelets and activated platelets, respectively. The mean fluorescence intensity was defined as the geometric mean calculated from a histogram of PE fluorescence per 10,000 FITC-positive cells.

### Collagen-induced thrombocytopenia

The rats were anaesthetised with sodium thiopental (100 mg/kg, i.p.). Collagen solution (collagen I, rat tail, A1048301, Thermo Fisher Scientific, Inc.) was diluted with physiological saline (saline, Otsuka Pharmaceutical Factory, Inc.) at concentrations of 0.1, 0.3, and 1 mg/mL. The saline served as a vehicle. The collagen solutions (1 mL/kg) or their vehicle were administered to the rats intravenously as a bolus to the jugular vein. Blood was collected from the jugular vein into a syringe containing 10 vol% of 3.8% sodium citrate before and 10, 30, 60, 120, and 180 min after collagen injection, and platelets were counted with an automated haematology analyser (Cellutac α, Nihon Kohden Corporation). The change in platelet count was expressed as the percentage change from before collagen injection, and the AUC of platelet count (% · min) was also calculated.

### Rat thrombosis model

Thrombus formation was induced in 6-week-old rats. The rats were anaesthetised with isoflurane, and the right carotid artery was exposed. A soft-cuff blood flow probe (MC0.5PSB, Transonic Systems, Inc.) was placed on the right carotid artery, and blood flow was continuously monitored with a pulsed Doppler flow metre (TS420, Transonic Systems, Inc.). Subsequently, filter paper (qualitative filter paper no. 2; Advantec Toyo Kaisha, Ltd.) trimmed to 2 × 3 mm and saturated with a 40% FeCl_3_ solution was placed on the arterial surface for 3 min. The filter paper was removed, and TTO, defined as the time to arrest blood flow for >1 min, was measured. Blood flow was monitored for 60 min after placement of the filter paper. If the blood flow was not arrested for >1 min during the 60 min observation, TTO was defined as 60 min, and monitoring was terminated.

The rats were euthanised by exsanguination under isoflurane anaesthesia. Perfusion fixation was performed with 10% neutral-buffered formalin for 5 to 10 min, and the FeCl_3_-injured regions of the right carotid arteries were collected and fixed in 10% neutral-buffered formalin. Specimens were isolated from three areas of the carotid arteries: approximately at the midsection of the FeCl_3_-injured region, 100 μm distal to the midsection, and 200 μm distal to the midsection. Paraffin sections were prepared, and each region was stained with HE and EVG. For EVG-stained specimens, the luminal area (A) and the thrombus area (T) were measured by image analysis software (Win ROOF 2013; Mitani Corporation), and lumen stenosis (100 × T/A) was calculated. The mean values of T and lumen stenosis in three positions were calculated.

### Rat bleeding model

The rats were anaesthetised with thiopental sodium (75 mg/kg, i.p.). After blinding of the experimenter, an incision was made (depth 1 mm) with a blade (No. 11, FEATHER Safety Razor Co., Ltd.) on the artery of the ventral part of the tail at 4 cm from the tip, and the blood was blotted every 15 s with filter paper (No. 2, Advantec Toyo Kaisha, Ltd.) for 30 min. The bleeding time was defined as the multiplication of the number of detectable blood stains on the opposite side of the filter paper that touched the blood by 15 s.

### PRI

VASP phosphorylation was determined according to the kit instructions for human samples (CY-QUANT VASP/P2Y12, BioCytex). Whole blood samples were preincubated with R-138727 (1, 3, 10, 30, and 100 μmol/L) or vehicle (saline) for 1 h. The blood samples were added to pairs of microtubes containing activator A (prostaglandin E1 [PGE_1_]) and activator B (PGE_1_ + ADP). Following incubation at room temperature for 10 min and lysis, the PGE_1_-activated samples, the corresponding PGE_1_ + ADP-activated samples, and a dilution buffer as a blank were loaded on a 96-well plate coated with mouse anti-human VASP monoclonal antibody, and the wells were covered, incubated for 30 min at room temperature, and washed three times with 300 μL of washing solution. Specific mouse anti-human phosphorylated-VASP monoclonal antibody coupled to peroxidase was added immediately. The wells were covered, incubated for 30 min at room temperature, and washed as above. 3,3ʹ,5,5′-Tetramethylbenzidine was added for color development, and the wells were incubated for a further 5 min at room temperature. The reaction was stopped by acidification with H_2_SO_4_, resulting in a yellow product with an absorbance peak at 450 nm. Absorbance at 450 nm was measured with a plate reader (SpectraMax Plus 384, Molecular Devices, LLC). Phosphorylated VASP antigen in the blood and PRI were calculated using the absorbance according to the formulas

$${\rm{Phosphorylated}}\,{\rm{VASP}}\,{\rm{antigen}}={\rm{OD}}\,({{\rm{PGE}}}_{1})-{\rm{OD}}\,({\rm{blank}})$$

and

$${\rm{PRI}}\,( \% )=\frac{\mathrm{OD}\,({{\rm{PGE}}}_{1})-\mathrm{OD}\,({{\rm{PGE}}}_{1}+{\rm{ADP}})}{\mathrm{OD}\,({{\rm{PGE}}}_{1})-\mathrm{OD}\,({\rm{blank}})}\times \,100$$

where OD (PGE_1_) = absorbance at 450 nm (activator A sample),

OD (PGE_1_ + ADP) = absorbance at 450 nm (activator B sample), and

OD (blank) = absorbance at 450 nm (dilution buffer, blank).

### Statistical analysis

Calculations other than IC_50_ determination were performed with Microsoft Excel 2013 (Microsoft Corporation). Data are expressed as means ± standard error of the mean (SEM). Statistical analyses of data for haematology, blood biochemistry, thrombogenesis, and haemostasis were performed by Student’s *t*-test (VASP^+/+^ vs. VASP^−/−^ as a primary analysis and VASP^+/−^ vs. VASP^−/−^ as a subanalysis), Dunnett’s multiple comparison test (PBS-treated group vs. ADP-treated groups per genotype in flow cytometry, VASP^+/+^ vs. VASP^+/−^ and VASP^−/−^ in phosphorylated VASP antigen, and vehicle vs. R-138727-treated groups in PRI and IPA), and Spearman’s rank correlation coefficient hypothesis testing (dose dependency of R-138727). All statistical analyses and calculation of IC_50_ values and 95% CIs were performed with SAS System Release 9.2 (SAS Institute, Inc.). *P* values of <0.05 were considered to indicate statistically significant differences.

## Electronic supplementary material

Supplementary information
