# Supplementary material for: Vasodilator-stimulated phosphoprotein (VASP) is not a major mediator of platelet aggregation, thrombogenesis, haemostasis, and antiplatelet effect of prasugrel in rats
Source: Sci Rep. 2018 Jul 2;8:9955. doi: 10.1038/s41598-018-28181-8 (PMC6028634; doi:10.1038/s41598-018-28181-8)
Supplement: Supplementary file 1 — Supplementary information [file 41598_2018_28181_MOESM1_ESM.pdf]

Supplementary information for:

Vasodilator-stimulated phosphoprotein (VASP) is dispensable for platelet aggregation, thrombogenesis, haemostasis, and antiplatelet effect of prasugrel in rats

Yusuke Ito, Kousaku Ohno, Yuka Morikawa, Atsuyuki Tomizawa, Makoto Mizuno, and Atsuhiro Sugidachi

Rare Disease & LCM Laboratories, Daiichi Sankyo Co., Ltd., Tokyo, Japan.

Corresponding author: Atsuhiro Sugidachi

Supplementary Figure S1

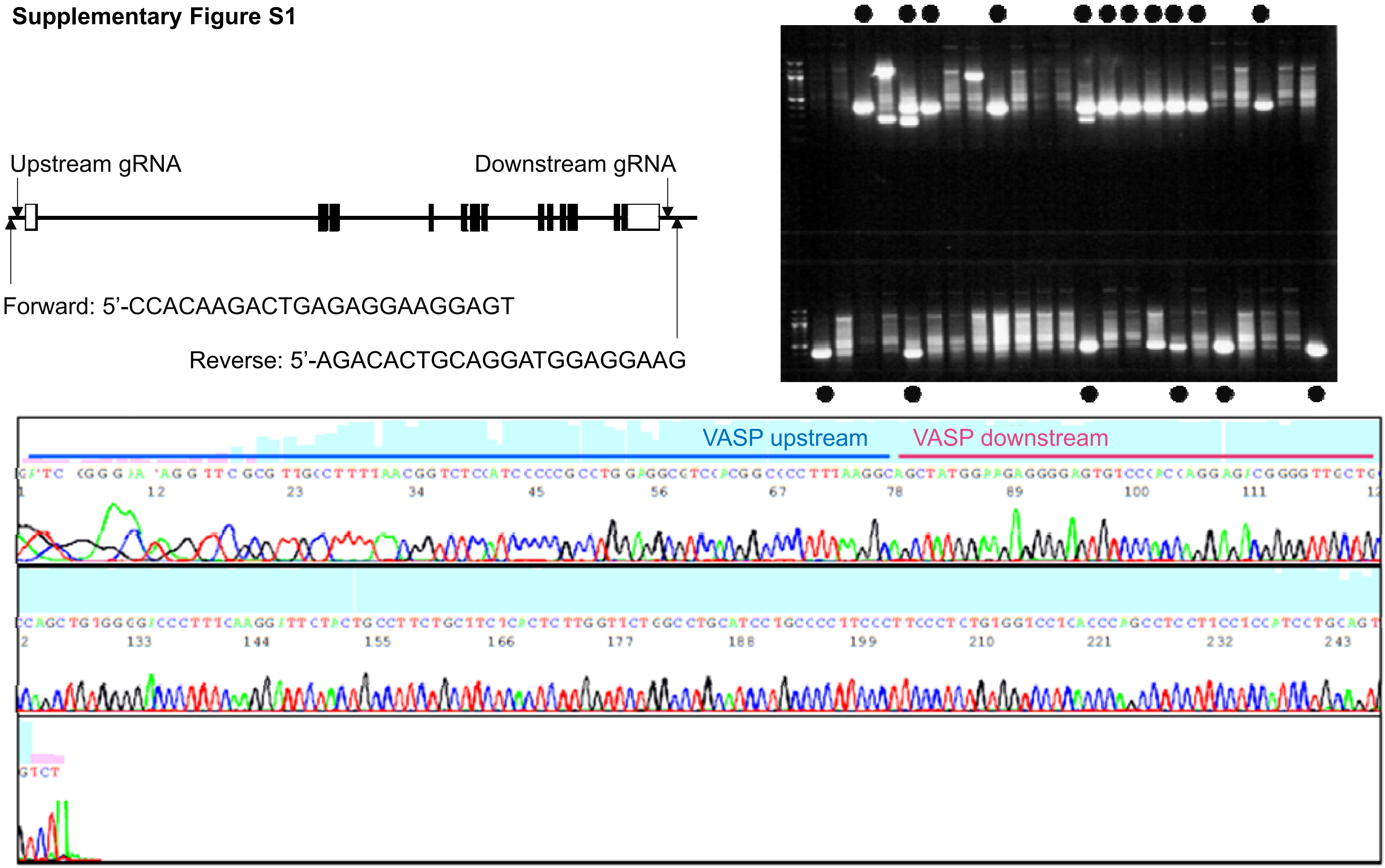

Supplementary Figure S1. Analysis of vasodilator-stimulated phosphoprotein (VASP) gene deletion in rats
The upper left panel shows a schematic illustration of VASP gRNAs and polymerase chain reaction (PCR) primers. The upper right panel shows a typical result of agarose gel electrophoresis of PCR products. The left extremity lanes are DNA ladder, and VASP deletion mutants (closed circles) brought smaller PCR products. Direct sequencing revealed entire deletion of the VASP gene in F1 rats (lower panel).

Supplementary Figure S2

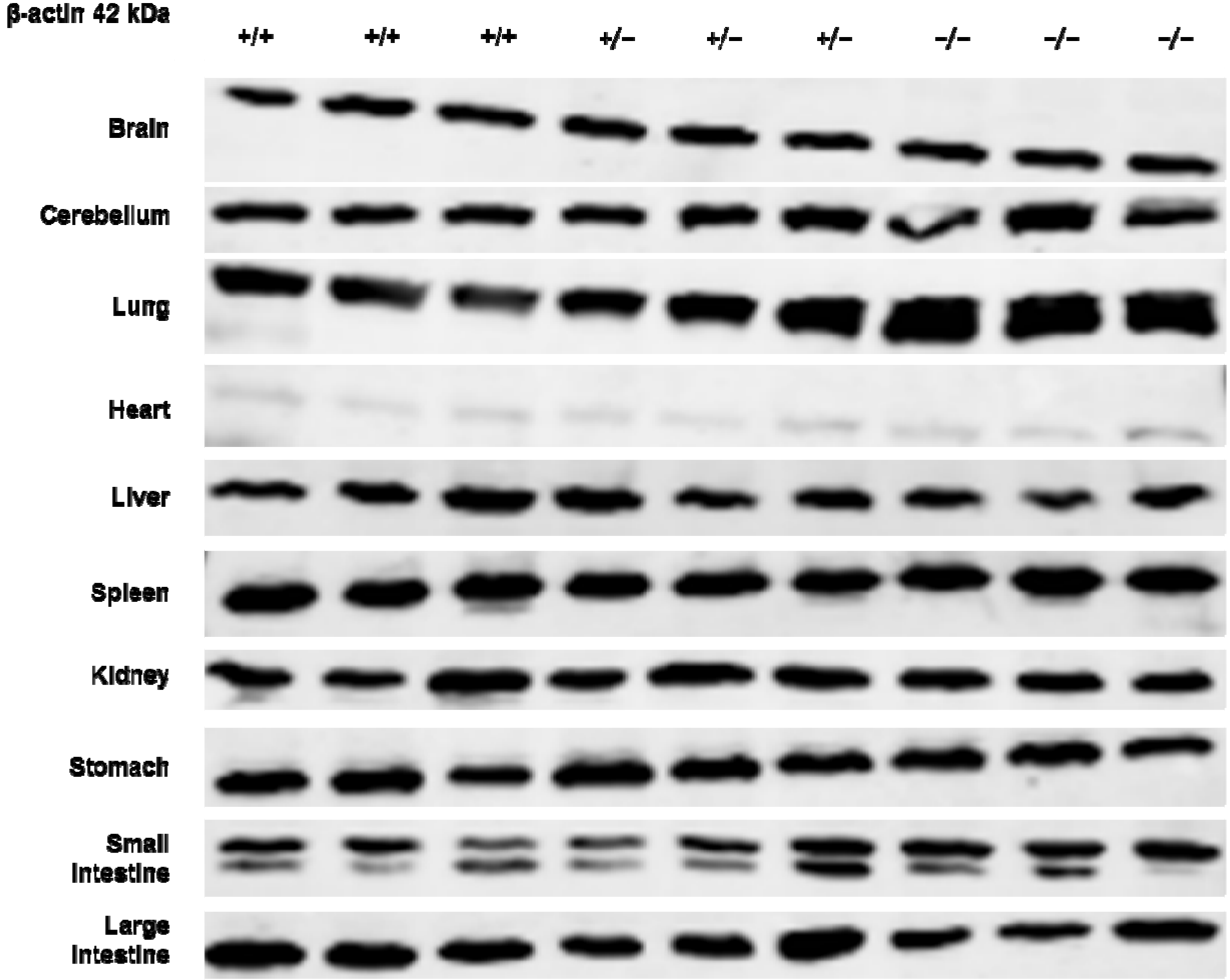

Supplementary Figure S2. β-actin expression in VASP$^{+/+}$, VASP$^{+/-}$, and VASP$^{-/-}$ rats

Sodium dodecyl sulfate polyacrylamide gel electrophoresis (SDS-PAGE) and Western blot analysis were performed using 1 μg of protein extracted from each organ. The primary antibody used for β-actin was monoclonal anti-β-actin antibody produced in mouse (A5441, Sigma-Aldrich Co. LLC) with 10,000-fold dilution. n = 3 per genotype. +/+, wild type; +/−, heterozygous VASP knockout; −/−, homozygous VASP knockout. VASP, vasodilator-stimulated phosphoprotein.

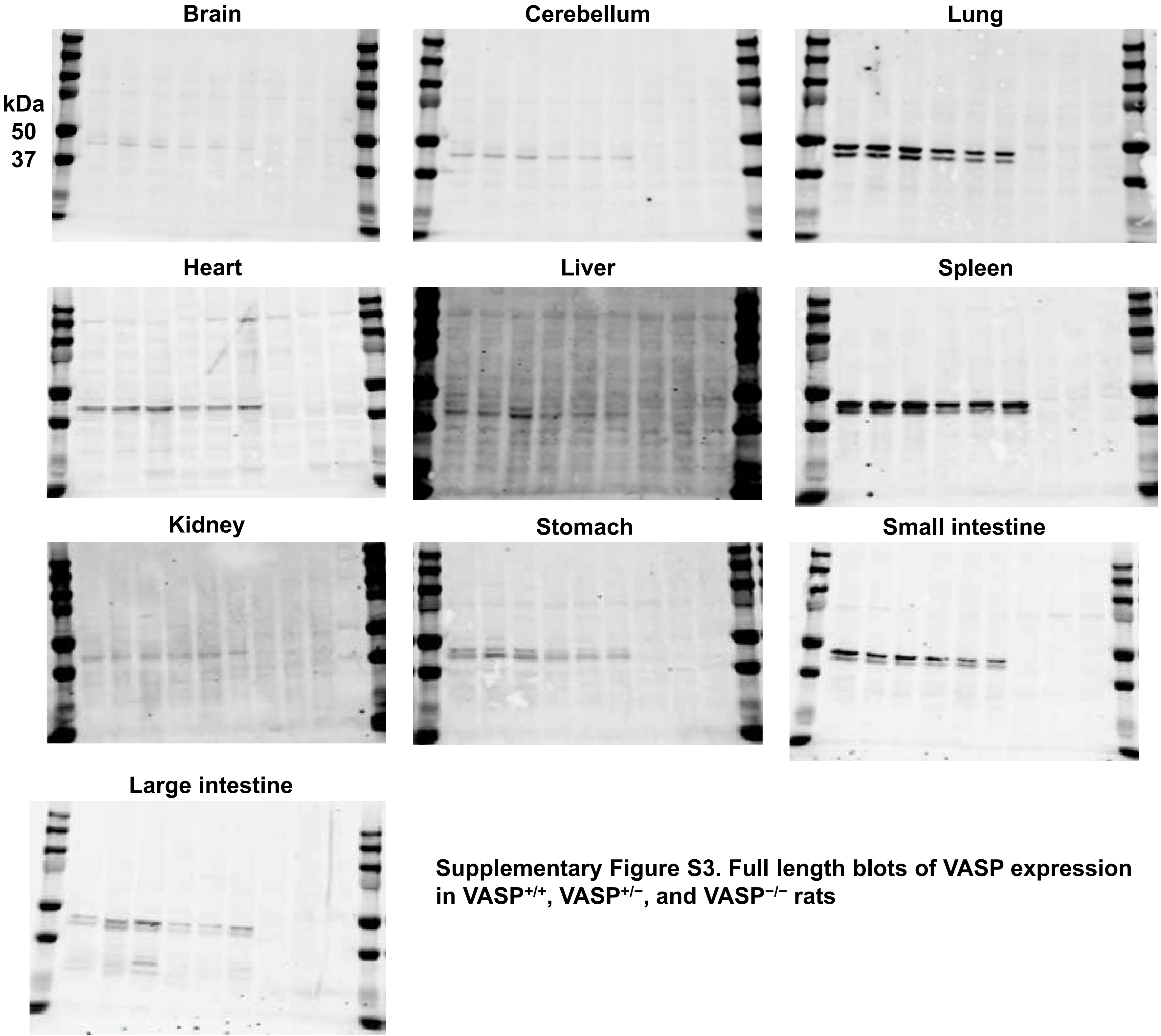

Supplementary Figure S3. Full length blots of VASP expression in VASP$^{+/+}$, VASP$^{+/-}$, and VASP$^{-/-}$ rats

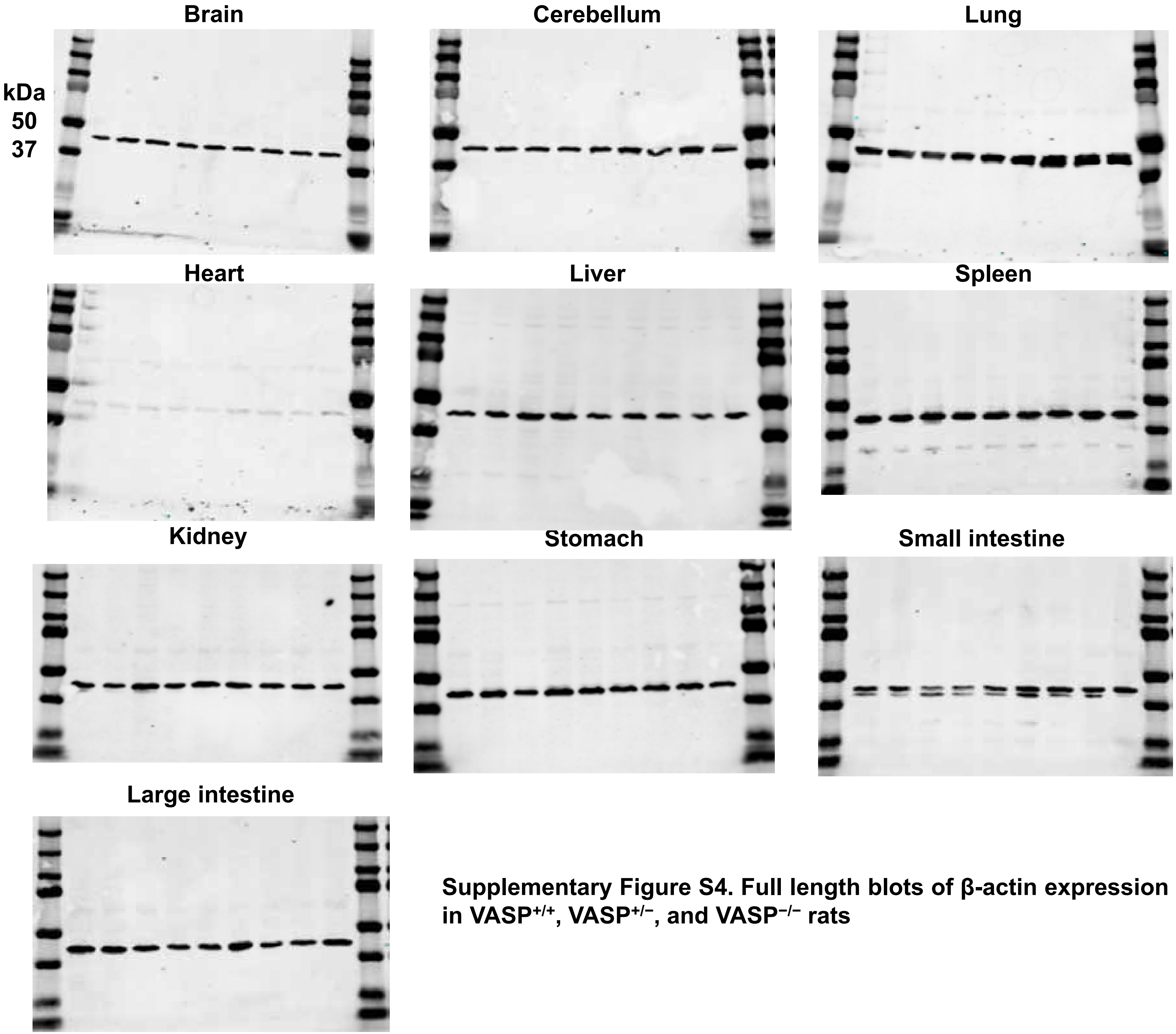

Supplementary Figure S4. Full length blots of β-actin expression in $VASP^{+/+}$, $VASP^{+/-}$, and $VASP^{-/-}$ rats

Supplementary Table S1 Candidate off-target genes in vasodilator-stimulated phosphoprotein (VASP) gRNAs

VASP upstream gRNA

Sequence: GTGCAGCGTCCGAACCTCGCCGG
on-target locus: chr1:-81452591
number of offtarget sites: 23 (9 are in genes)

Off-target candidate sequences	Score	Mismatches	UCSC gene	Locus	Gene symbol
GTCCAGCCTCGGAACCTCGGCAG	0.4	4MMs [3:8:11:20]	NM_001108335	chr12:-43306462	Rasal1
CTGCAGCGTGAGCACCTCGCGGG	0.3	4MMs [1:10:11:13]	NM_001106443	chr2:+207615574	Msto1
GTTCAGCCTCCGAACCACGTCGG	0.3	4MMs [3:8:17:20]	NM_001025741	chr6:-141552861	Eml1
GTGCTGCGTCTCAACCTCTCTGG	0.1	4MMs [5:11:12:19]	NM_001109128	chr19:+48090791	Fbxl8
CTGCAGCCTCCGCACCTGGCCAG	0.1	4MMs [1:8:13:18]	NM_001270947	chr3:-157283350	Tp53inp2

VASP downstream gRNA

Sequence: TAAATGCTATGCCCCATCGAAGG
on-target locus: chr1:+81437056
number of offtarget sites: 90 (6 are in genes)

Off-target candidate sequences	Score	Mismatches	UCSC gene	Locus	Gene symbol
CTAATGCTTTGCCCCATCCAAAG	0.3	4MMs [1:2:9:19]	NM_001033923	chr4:+142950824	Gimap8
TACATTCAATGCCCCATGGATGG	0.2	4MMs [3:6:8:18]	NM_001034949	chr5:+161211217	Tmco4
TAAATGCTATGCCAGTTCCATGG	0	4MMs [14:15:16:19]	NM_001170329	chr1:-82806734	RGD1564380
TAAATGCTATGCCAGTTCCATGG	0	4MMs [14:15:16:19]	NM_001170329	chr1:-82813467	RGD1564380
TAAATGCTATGCCAGTTCCATGG	0	4MMs [14:15:16:19]	NM_001170329	chr1:-84155999	RGD1564380

The number of possible off-target sites was 23 (9 in genes) for upstream and 90 (6 in genes) for downstream.
The top five candidate genes are listed.

Supplementary Table S2 Primer pairs of off-target candidates

Gene symbol		
Rasal1	Forward	5'-AGATCGCAGCGCACAGCTAC
	Reverse	5'-CTGAGAGCCCCCTCCCACAG
Msto1	Forward	5'-GGCCCTAGAGGAGGAAGAGG
	Reverse	5'-AAGACGTCCGGGCACAGCTC
EMl1	Forward	5'-TCTTAGGTCGGTAGACTGTGTCG
	Reverse	5'-CAAAGCACACCAACAAAGACTGT
Fbxl8	Forward	5'-CCCTTTTACACTTCTGGCACTG
	Reverse	5'-CCAGTACAGAGGGTCTCACCAC
Tp53inp2	Forward	5'-TGTACCCCTCAGTTAGGACATCA
	Reverse	5'-CTGCTACAGAGGAGGCAGAAGG
Gimap8	Forward	5'-CACAAAGGAAACTGAGGACAGTG
	Reverse	5'-GCTCCATAGAACAACAGAGTCCA
Tmco4	Forward	5'-TTTCACACAGAGGGAGTAGACCA
	Reverse	5'-CCAGGCTACCTTGGATTACAAAC
RGD1564380	Forward	5'-CAGCACCAAAGTGCTACTTGACT
	Reverse	5'-ATTCCTCCCTAACACCCAGAGTT
